# Comprehensive top-down mass spectral repository enables pan-dataset analysis and top-down spectral prediction

**DOI:** 10.64898/2026.02.20.707032

**Published:** 2026-02-23

**Authors:** Kun Li, Kaiyuan Liu, James M. Fulcher, Haixu Tang, Xiaowen Liu

## Abstract

Mass spectral libraries have become essential resources for training deep learning (DL) models for spectral prediction and *de novo* sequencing in bottom-up mass spectrometry (BU-MS). Compared with BU-MS, top-down MS (TD-MS) offers unique advantages for characterizing intact proteoforms by analyzing proteoforms without enzymatic digestion. Despite these advantages, large-scale spectral libraries for TD-MS are currently lacking. Here we present TopRepo, the first comprehensive repository of TD-MS spectra, comprising more than 18 million spectra acquired from 12 species across eight types of mass spectrometers. Using TopRepo, we constructed a large-scale top-down spectral library containing over 5 million spectra with curated proteoform and fragment-ion annotations. We demonstrate that TopRepo enables pan-dataset analyses of N-terminal processing, mass shifts, and other proteoform characteristics identified by TD-MS. Furthermore, we show that the TopRepo spectral library substantially improves proteoform identification through spectral library searching and supports the training of DL models for high-accuracy top-down spectral prediction.

## Full Text Availability

The license terms selected by the author(s) for this preprint version do not permit archiving in PMC. The full text is available from the preprint server.

